# Sexual function and its associations with anxiety, depression, functionality, and quality of life in women with lipedema

**DOI:** 10.1093/sexmed/qfag041

**Published:** 2026-06-07

**Authors:** Hatice Betigül Meral, Sena Tolu, Bengisu Meral Ketenci, Zeynep Noyan, Aylin Rezvani

**Affiliations:** Istanbul Medipol University, Faculty of Medicine, Department of Physical Medicine and Rehabilitation, 34214 Istanbul, Türkiye; Istanbul Medipol University, Faculty of Medicine, Department of Physical Medicine and Rehabilitation, 34214 Istanbul, Türkiye; Department of Dermatology, Konya City Hospital, 42020 Konya, Türkiye; Istanbul Medipol University, Faculty of Medicine, Department of Physical Medicine and Rehabilitation, 34214 Istanbul, Türkiye; Istanbul Medipol University, Faculty of Medicine, Department of Physical Medicine and Rehabilitation, 34214 Istanbul, Türkiye

**Keywords:** sexual dysfunction, lipedema, quality of life, depression, anxiety

## Abstract

**Background:**

Lipedema is a chronic adipose tissue disorder primarily affecting women, characterized by abnormal fat accumulation, pain, and reduced mobility. Its impact on sexual function remains underexplored. This study aimed to evaluate sexual function in women with lipedema and examine its associations with anxiety, depression, lower extremity function, and quality of life.

**Methods:**

In this cross-sectional study, 100 sexually active women were recruited: 50 with lipedema and 50 age- and Body Mass Index-matched healthy controls. Sexual function was assessed with the Female Sexual Function Index (FSFI); anxiety and depression with the Hospital Anxiety and Depression Scale (HADS-A and HADS-D); quality of life with the EuroQOL 5-Dimensional 5-Level (EQ-5D-5L) instrument; lower extremity function with the Lower Extremity Functional Scale (LEFS); and pain intensity with the Visual Analog Scale (VAS). Multiple linear regression analysis was conducted to identify the factors associated with the total FSFI score.

**Results:**

Women with lipedema had significantly lower total FSFI scores compared to controls (21.58 ± 3.99 vs. 25.86 ± 3.21, *P* < .001), with 76% having FSFI scores below the cut-off (≤ 26.55) compared to 36% of controls. All FSFI domain scores were significantly lower in the lipedema group (all *P* < .05). In the lipedema group, there was a significant correlation between total FSFI scores and age (*P* = .002), pain intensity (VAS; *P* = .022), depression (HADS-D; *P* = .010), quality of life (EQ-5D-5L index; *P* = .027), and lower extremity function (LEFS; *P* < .001). Multiple linear regression analysis identified depression (HADS-D; *P* = .047), perceived health status (EQ-5D-5L VAS; *P* = .033), and lower extremity function (LEFS; *P* = .011) as independent variables that had a significant relationship with the total FSFI score.

**Discussion:**

Lower sexual function is common among women with lipedema and is associated with anxiety and depressive symptoms, lower extremity function, and pain intensity. These findings highlight the importance of incorporating sexual function assessment into the routine evaluation of patients with lipedema and support the need for comprehensive multidisciplinary treatment approaches addressing physical, psychological, and sexual health aspects of care.

## Introduction

Lipedema is a chronic and progressive adipose tissue disorder that predominantly affects women. First described by Allen and Hines in 1940, lipedema is characterized by the symmetrical accumulation of pathologic subcutaneous fat, primarily in the lower extremities and sometimes the arms, sparing the hands and feet.^1^ Although its prevalence is estimated to be as high as 11% among women globally, lipedema remains underdiagnosed due to overlapping symptoms with obesity, lymphedema, and chronic venous insufficiency.^2-4^ The condition often develops or worsens during periods of hormonal change, such as puberty, pregnancy, or menopause, suggesting a hormonal and potentially genetic etiology.^5^^,^^6^

Clinically, patients present with chronic leg pain, sensitivity to touch, easy bruising, and disproportionate fat distribution in the lower body that is resistant to diet and exercise.^7^ In advanced stages, mobility becomes increasingly impaired due to joint stress, tissue fibrosis, and musculoskeletal dysfunction.^8^

Lipedema has been associated with a reduced quality of life in women from early adulthood, both through physical limitations and challenges in social interactions. In recent years, it has increasingly been recognized that this condition may also be related to aspects of sexual functioning and intimate relationships, beyond physical health. Sexual functioning is a multidimensional and complex aspect of health, conceptualized within a biopsychosocial framework encompassing domains such as desire, arousal, orgasm, satisfaction, and pain.^9^ Within this framework, sexual desire refers not only to the motivation or interest in sexual activity but also to cognitive and behavioral components related to sexual stimuli; arousal involves both physiological responses and subjective experiences; orgasm is defined as the peak of sexual pleasure accompanied by coordinated neurophysiological processes; satisfaction reflects the individual’s cognitive and emotional appraisal of sexual experiences; and pain refers to discomfort associated with sexual activity, which may include both physiological and psychological components.^9^^,^^10^ Sexual functioning is associated with factors related to physical comfort, body image, emotional balance, and interpersonal relationships.^10-12^ In individuals with lipedema, negative body image, sociocultural pressure to conform to beauty ideals, and decreased self-esteem have been discussed as potential psychosocial factors.^13^ Negative body image has been associated with lower self-esteem and dissatisfaction in one’s relationship with oneself and others, and may influence sexual confidence, thereby shaping behaviors related to sexuality.^14^ Furthermore, in lipedema, the chronic nature of the disease, along with the accompanying emotional burden and social stigma, may also be associated with strain in romantic relationships and reduced quality of sexual life.^15^^,^^16^

Lower extremity function plays a critical role not only in physical independence but also in psychological well-being and sexual function.^17^ In women with lipedema, leg pain, reduced muscle strength, and limited mobility may restrict participation in social life and negatively affect daily functioning, potentially contributing to the emergence of psychological symptoms such as anxiety and depression.^18^ Accordingly, lipedema may influence sexual function both through direct impairment of physical functioning and indirectly through these psychological symptoms; this interaction may contribute to a cycle that exacerbates symptom severity and reduces quality of life. Despite growing awareness of these associations, knowledge remains limited regarding the prevalence, underlying causes, and associated risk factors of sexual dysfunction in women with lipedema. The evaluation of sexual function in individuals with lipedema represents a critical step not only in recognizing the multidimensional impact of the disease but also in supporting a more holistic approach to its management and improving overall quality of life. Accordingly, this study aimed to evaluate sexual function in women with lipedema, compared with age- and body mass index-matched healthy controls, and to examine its associations with anxiety, depression, pain intensity, lower extremity function, and quality of life. We hypothesized that women with lipedema would have lower sexual function scores than healthy controls. The study was designed to address the following research question: Do women with lipedema exhibit lower sexual function compared to matched healthy controls, and is there a relationship between sexual function and anxiety, depression, pain intensity, lower extremity function, and quality of life in this population?

## Materials and methods

### Design and participants

This cross-sectional study included a total of 100 female participants. The patient group comprised 50 individuals who presented to the Physical Medicine and Rehabilitation outpatient clinic of our hospital between September and December 2025 and were diagnosed with lipedema after clinical evaluation. The control group consisted of 50 age- and body mass index (BMI)-matched healthy controls who were recruited from individuals presenting to the hospital for routine annual health check-ups without any known medical conditions. All participants in the control group underwent clinical evaluation by physicians and did not report lipedema-associated symptoms or show any clinical signs of the disease. The recruitment period lasted approximately 3.5 months.

Lipedema was diagnosed by experienced physicians according to pre-defined clinical criteria, characterized by bilateral and symmetrical fat accumulation in the limbs with little or no involvement of the hands and feet, poor response to weight reduction efforts (excluding ketogenic diets), presence of pain, tenderness, easy bruising, sensitivity or fatigue in the affected areas, minimal or absent pitting oedema, and lack of symptom relief with limb elevation.^19-21^

Inclusion criteria for both groups were: biologically female sex, beyond the age of 18, being sexually active. Exclusion criteria for both groups included pregnancy or breastfeeding, active malignancy, use of medications that could influence mood or sexual function (such as selective serotonin reuptake inhibitors, antipsychotics, estrogen and/or androgen therapy, beta-blockers, etc.), cognitive impairment that could interfere with questionnaire completion, and a BMI greater than 50 kg/m^2^, as this level of obesity is associated with systemic health complications. Additionally, individuals with previously impaired sexual function due to genital disorders, musculoskeletal system disorders, neurological or psychiatric disorders, diabetes, cardiovascular diseases, prior surgery, or radiotherapy involving the hip were excluded.

Patients with other types of edema with a non-lipedema component—such as lymphedema, venous edema, or edema secondary to cardiac or renal failure—were also excluded.

### Ethical considerations

Institutional ethics committee approval was obtained before the study, and written informed consent was obtained from all participants. All questionnaires were administered in paper-based format during face-to-face interviews conducted in a clinical setting. Participants completed the questionnaires under the supervision of a trained researcher to ensure completeness and to minimize missing or inconsistent responses. No financial compensation or incentives were provided to the participants.

### Demographic and clinical questionnaire

All participants completed a standardized data collection form that included demographic variables such as age, height, weight, marital status, educational status, and occupation. Anthropometric measurements were used to calculate BMI, which was classified according to the World Health Organization (WHO) criteria: normal weight (18–24.9 kg/m^2^), overweight (25–29.9 kg/m^2^), obesity class I (30–34.9 kg/m^2^), class II (35–39.9 kg/m^2^), and class III (≥40 kg/m^2^).^22^

In addition to demographic data, participants in the lipedema group provided detailed clinical information, including disease type, clinical stage, lipedema onset (eg, puberty, pregnancy), disease duration (years), and family history of lipedema.

Lipedema types were categorized into five groups based on anatomical distribution: type I involving the pelvis and buttocks; type II involving the region from the pelvis to the knees; type III characterized by fat accumulation from the hips to the ankles with a visible “cuff sign” at the ankle, sparing the feet; type IV involving the upper limbs from the shoulders to the wrists; and type V predominantly affecting the calves.^23^ The clinical stage of lipedema was determined by assessing the characteristics of subcutaneous fat and skin texture. Stage 1 was defined by smooth skin over a thickened, nodular hypodermis; stage 2 by uneven skin with palpable, pearl-sized nodules, often described as having an “orange peel” appearance; stage 3 by lobular protrusions of fat and connective tissue that cause visible deformities, particularly around the thighs and knees; and stage 4 by the coexistence of lymphoedema.

The pain severity of the participants was assessed using the Visual Analog Scale (VAS), a validated 10-cm horizontal scale ranging from 0 (no pain) to 10 (worst imaginable pain). After receiving standardized verbal instructions regarding the scale’s use, each participant was asked to indicate their current level of pain by placing a mark on the line.^24^

Sexual function was assessed using the Female Sexual Function Index (FSFI), a validated self-report questionnaire designed to evaluate female sexual function across six domains: desire, arousal, lubrication, orgasm, satisfaction, and pain. Each domain is scored using a 6-point Likert-type scale, and domain scores are summed to obtain a total FSFI score, with lower scores indicating poorer sexual function. A total score of 26.55 or below was considered indicative of sexual dysfunction. The Turkish version of the FSFI, validated by Aygin and Aslan, demonstrated internal consistency coefficients ranging from 0.70 to 0.90 and a Cronbach’s alpha value of 0.98.^25^ In the present study, the FSFI demonstrated high internal consistency, with a Cronbach’s alpha coefficient of 0.96.

Health-related quality of life was assessed using the five-level version of the EuroQol Group’s EQ-5D instrument (EQ-5D-5L). It includes five dimensions: mobility, self-care, usual activities, pain/discomfort, and anxiety/depression.^26^ Each dimension is rated on a 5-level scale ranging from “no problems” to “extreme problems.” In addition, the EQ-5D-5L incorporates a VAS, where participants rate their overall health from 0 (worst imaginable health) to 100 (best imaginable health). The Turkish version of the scale, translated by the EuroQol Group, was used in this study and has been reported to have Cronbach’s alpha values above 0.80. In the present study, the EQ-5D-5L demonstrated acceptable internal consistency, with a Cronbach’s alpha coefficient of 0.78.

Anxiety and depressive symptoms were evaluated using the Hospital Anxiety and Depression Scale (HADS). The instrument comprises 14 items, with seven items assessing anxiety (HADS-A) and seven assessing depression (HADS-D). Responses are scored on a four-point Likert scale ranging from 0 to 3, yielding subscale scores between 0 and 21. Higher scores reflect greater symptom severity. The Turkish version of the HADS has been validated by Aydemir et al. with Cronbach’s alpha values reported as 0.85 for the anxiety subscale and 0.78 for the depression subscale.^27^ In the present study, the HADS demonstrated good internal consistency, with Cronbach’s alpha coefficients of 0.83 for HADS-A and 0.81 for HADS-D.

Lower extremity function was assessed using the Lower Extremity Functional Scale (LEFS), a validated self-reported questionnaire consisting of 20 items, each rated on a 5-point scale (0–4), reflecting increasing levels of functional ability. Total scores range from 0 to 80, with higher scores indicating better function. The Turkish version of the LEFS was used in this study and has been shown to have a Cronbach’s alpha value of 0.92.^28^ In the present study, the LEFS demonstrated high internal consistency, with a Cronbach’s alpha coefficient of 0.93.

### Statistical analysis

Descriptive statistics were used to summarize the continuous variables, including mean and standard deviation. The normality of the data was assessed using the Shapiro–Wilk test. For comparisons between two independent groups, the Student’s *t-*test was applied for normally distributed variables, whereas the Mann–Whitney U test was used for non-normally distributed variables. Categorical variables were presented as numbers and percentages, and comparisons were conducted using Fisher’s Exact test. The relationships between continuous variables that did not follow a normal distribution were analyzed using Spearman’s rho correlation coefficients. To determine the factors associated with the total FSFI score, a multiple linear regression analysis was performed using the backward variable selection method. Multicollinearity was assessed using variance inflation factor. To account for the variability introduced by the backward variable selection procedure, a bootstrap variable selection analysis was performed using 5000 bootstrap samples. In each bootstrap sample, the full set of candidate predictors was entered into the model, the backward elimination procedure was repeated, and the selected variables were recorded. Selection frequencies were calculated as the percentage of bootstrap samples in which each predictor was retained. The Durbin–Watson statistic was calculated to evaluate the presence of autocorrelation in the residuals. Statistical significance was set at *P* < .05. Data analysis was performed using IBM SPSS Statistics, Version 24.0 (IBM Corp., Armonk, NY, USA).

A post hoc power analysis was performed based on the primary outcome (total FSFI score) using the observed between-group difference. The calculated effect size (Cohen’s d) was 1.18, corresponding to a post hoc statistical power of greater than 99% at a two-sided significance level of 0.05.

## Results

A total of 100 women were included in the study: 50 in the lipedema group and 50 in the control group. The groups were similar in age and BMI, with no significant differences (*P* > .05). Among women with lipedema, the disease most frequently began during puberty (38%), and the majority of patients were classified as lipedema Type II (50%) and Stage 2 (52%). The mean disease duration was 14.66 ± 9.49 years, and 56% of patients reported a family history of lipedema. Detailed demographic and clinical characteristics of the participants are presented in Table 1.

**Table 1:** Demographic and clinical characteristics of study participants (*n* = 100).

	Lipedema group (*n* = 50)	Control group (*n* = 50)	*P* value
**Age (years), Mean (SD)**	41.28 ± 6.59	39.58 ± 6.15	.169^a^
**BMI (kg/m** ^2^ **), Mean (SD)**	32.34 ± 5.17	30.77 ± 4.63	.113^b^
**BMI classification, N (%)**			
Normal weight (18.5–24.9)	4 (8)	8 (16)	.526^c^
Overweight (25–29.9)	12 (24)	12 (24)	
Obesity class I (30–34.9)	19 (38)	21 (42)	
Obesity class II (35–39.9)	10 (20)	7 (14)	
Obesity class III (≥40)	5 (10)	2 (4)	
**Educational status, N (%)**			
Without formal education	2 (4)	1 (2)	.967^c^
Primary school	13 (26)	13 (26)	
Secondary school	5 (10)	7 (14)	
High school	11 (22)	10 (20)	
University	19 (38)	19 (38)	
**Occupation, N (%)**			
Homemaker	23 (46)	15 (30)	.221^c^
Officer	19 (38)	23 (46)	
Worker	8 (16)	12 (24)	
**Lipedema onset, N (%)**			
Childhood	3 (6)		
Puberty	19 (38)		
Pregnancy	14 (28)		
Menopause	4 (8)		
Not specified	10 (20)		
**Disease duration (years), Mean (SD)**	14.66 ± 9.49		
**Family history of lipedema, N (%)**			
Yes	28 (56)		
No	22 (44)		
**Lipedema types, N (%)**			
Type I	10 (20)		
Type II	25 (50)		
Type III	15 (30)		
**Lipedema stages, N (%)**			
Stage 1	6 (12)		
Stage 2	26 (52)		
Stage 3	18 (36)		

Abbreviations: SD = Standard Deviation; BMI = Body Mass Index.

a Mann–Whitney U test.

b Student t test.

c Fisher’s Exact test.

The total FSFI score was significantly lower in the lipedema group compared to the control group (21.58 ± 3.99 vs. 25.86 ± 3.21, *P* < .001), and 76% of women with lipedema had FSFI scores below the cut-off (≤ 26.55), compared to 36% of controls (*P* < .001) (Table 2).

**Table 2:** Comparison of clinical outcomes between women with lipedema and healthy controls.

	**Lipedema group (n = 50)**	**Control group (*n* = 50)**	***P* value**
**VAS score**	6.01 ± 1.60	-	-
**FSFI**			
Total	21.58 ± 3.99	25.86 ± 3.21	**<.001** ^**a**^
Desire	3.00 ± 0.90	3.89 ± 0.63	**<.001** ^**a**^
Arousal	3.49 ± 0.78	4.22 ± 0.52	**<.001** ^**a**^
Lubrication	4.10 ± 0.97	4.82 ± 0.75	**<.001** ^**a**^
Orgasm	3.42 ± 0.90	4.23 ± 0.70	**<.001** ^**a**^
Satisfaction	3.42 ± 1.00	4.28 ± 0.88	**<.001** ^**a**^
Pain	4.14 ± 0.70	4.41 ± 0.73	**.031** ^**a**^
**Sexual dysfunction, N (%)**			
Yes	38 (76)	18 (36)	**<.001** ^**b**^
No	12 (24)	32 (64)	
**HADS-A**	8.30 ± 2.57	5.20 ± 1.68	**<.001** ^**a**^
**HADS-D**	8.22 ± 2.74	3.80 ± 1.60	**<.001** ^**a**^
**EQ-5D-5L Index score**	0.80 ± 0.07	0.92 ± 0.07	**<.001** ^**a**^
**EQ-5D-5L VAS**	67.80 ± 12.13	87.06 ± 9.47	**<.001** ^**a**^
**LEFS**	56.18 ± 11.73	64.70 ± 4.73	**<.001** ^**a**^

Abbreviations: VAS **=** Visual Analog Scale; FSFI = Female Sexual Function Index; HADS-A = Hospital Anxiety and Depression Scale–Anxiety subscale; HADS-D = Hospital Anxiety and Depression Scale–Depression subscale; EQ-5D-5L = EuroQol 5 Dimensions, 5 Levels questionnaire; LEFS = Lower Extremity Functional Scale.

The data are shown as mean ± standard deviation, n, %.

Significant *P* values are written in bold.

a Mann–Whitney U test.

b Fisher’s Exact test.

**Figure 1:**
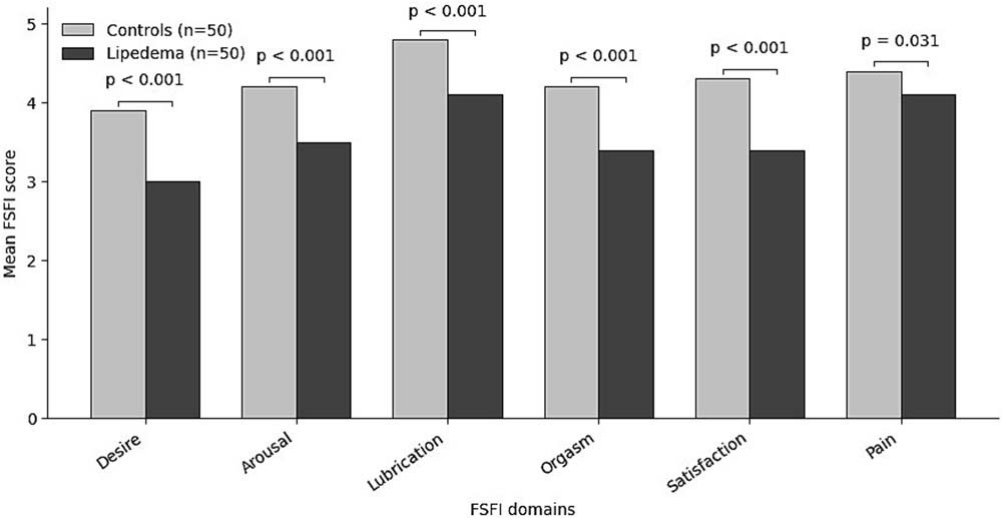
Comparison of mean FSFI domain scores between women with lipedema and healthy controls.

All FSFI domain scores were significantly lower in the lipedema group (all *P* < .05; Figure 1). The VAS score for pain in the lipedema group was 6.01 ± 1.60. Anxiety and depression scores were significantly higher in the lipedema group compared to the control group (HADS-A: 8.30 ± 2.57 vs. 5.20 ± 1.68; HADS-D: 8.22 ± 2.74 vs. 3.80 ± 1.60; *P* < .001 for both) (Table 2).

The EQ-5D-5L index score was lower in the lipedema group (0.80 ± 0.07 vs. 0.92 ± 0.07, *P* < .001), as was the EQ-5D-5L VAS score (67.80 ± 12.13 vs. 87.06 ± 9.47, *P* < .001). In addition, LEFS scores were significantly lower in the lipedema group compared to controls (56.18 ± 11.73 vs. 64.70 ± 4.73, *P* < .001) (Table 2).

In the lipedema group, the total FSFI score was significantly negatively correlated with age (*P* = .002), VAS score (*P* = .022), and HADS-D (*P* = .010), and positively correlated with EQ-5D-5L index score (*P* = .027) and LEFS (*P* < .001) (Table 3).

**Table 3:** Correlation analysis of FSFI total score and its domain scores with demographic and clinical variables in women with lipedema.

**Variables**		**Desire**	**Arousal**	**Lubrication**	**Orgasm**	**Satisfaction**	**Pain**	**Total FSFI**
Age (years)	*r =*	-0.430^**^	-0.361^*^	-0.423^**^	-0.128	-0.280^*^	-0.203	-0.429^**^
	*p =*	**0.002**	**0.010**	**0.002**	0.375	**0.049**	0.158	**0.002**
BMI (kg/m^2^)	*r =*	-0.020	0.068	-0.080	-0.140	0.011	-0.157	-0.088
	*p =*	0.888	0.638	0.582	0.332	0.940	0.277	0.542
VAS score	*r =*	-0.137	-0.357^*^	-0.175	-0.361^*^	-0.263	-0.210	-0.322^*^
	*p =*	0.342	**0.011**	0.224	**0.010**	0.065	0.144	**0.022**
Disease duration								
(years)	*r =*	-0.170	-0.033	-0.276	-0.130	-0.104	0.005	-0.157
	*p =*	0.238	0.820	0.052	0.369	0.471	0.974	0.277
Lipedema stages	*r =*	0.166	0.147	-0.074	-0.134	-0.071	-0.266	-0.038
	*p =*	0.249	0.309	0.608	0.355	0.625	0.062	0.792
HADS-A	*r =*	-0.031	-0.136	-0.086	-0.162	-0.293^*^	-0.301^*^	-0.080
	*p =*	0.832	0.345	0.551	0.262	**0.039**	**0.034**	0.582
HADS-D	*r =*	-0.291^*^	-0.381^**^	-0.191	-0.180	-0.362^**^	-0.135	-0.360^*^
	*p =*	**0.040**	**0.006**	0.184	0.210	**0.010**	0.350	**0.010**
EQ-5D-5L Index score	*r =*	0.461^**^	0.284^*^	0.333^*^	0.194	0.296^*^	-0.028	0.313^*^
	*p =*	**0.001**	**0.046**	**0.018**	0.177	**0.037**	0.847	**0.027**
EQ-5D-5L VAS	*r =*	0.143	0.241	0.169	0.170	0.043	0.061	0.137
	*p =*	0.323	0.092	0.242	0.239	0.767	0.672	0.344
LEFS	*r =*	0.266	0.343^*^	0.468^**^	0.300^*^	0.465^**^	0.323^*^	0.489^**^
	*p =*	0.062	**0.015**	**0.001**	**0.034**	**0.001**	**0.022**	**<0.001**

Abbreviations: BMI = Body Mass Index; VAS = Visual Analog Scale; FSFI = Female Sexual Function Index; HADS-A = Hospital Anxiety and Depression Scale–Anxiety subscale; HADS-D = Hospital Anxiety and Depression Scale–Depression subscale; EQ-5D-5L = EuroQol 5 Dimensions, 5 Levels questionnaire; LEFS **=** Lower Extremity Functional Scale.

Values of *P* < .05 were accepted as significant and are marked in bold.

^*^ *P* < .05, ^**^P < .01.

*r:* Spearman correlation coefficient.

At the domain level, FSFI desire was negatively correlated with age (*P* = .002) and HADS-D (*P* = .040), and positively correlated with EQ-5D-5L index score (*P* = .001). FSFI arousal was negatively correlated with age (*P* = .010), VAS score (*P* = .011), and HADS-D (*P* = .006), and positively correlated with LEFS (*P* = .015). FSFI satisfaction was negatively correlated with HADS-A (*P* = .039) and HADS-D (*P* = .010), and positively correlated with LEFS (*P* = .001). All other correlations are presented in Table 3.

In the lipedema group, a multiple linear regression analysis was performed to identify factors associated with the total FSFI score. Age, BMI, VAS score, disease duration, lipedema stage, HADS-A, HADS-D, EQ-5D-5L index score, EQ-5D-5L VAS, and LEFS were included as independent variables. The backward elimination method was applied. The final model revealed that HADS-D (B = −0.391, *t* = –2.040, *P* = .047), EQ-5D-5L VAS (B = 13.702, *t* = 2.203, *P* = .033), and LEFS (B = 0.106, *t* = 2.665, *P* = .011) were statistically significant predictors of the total FSFI score (Table 4).

**Table 4:** Multiple linear regression analysis of factors associated with total FSFI score in women with lipedema.

**Variant**	**B**	**Standard** **error**	**Beta**	***t*-value**	***P*-value**	**95%** **CI for B**	**VIF**	**Bootstrap selection frequency** ^**a**^ **(%)**	
						Lower	Upper		
Constant term (intercept)	16.874	7.247		2.328	**.025**	2.268	31.479		
BMI (kg/m^2^)	-0.173	0.094	-0.224	-1.838	.073	-0.362	0.017	1.42	39.6
VAS score	-0.558	0.302	-0.224	-1.843	.072	-1.167	0.052	1.58	51.2
HADS-D	-0.391	0.192	-0.268	-2.040	**.047**	-0.777	-0.005	1.76	55.9
EQ-5D-5L VAS	13.702	6.221	0.256	2.203	**.033**	1.165	26.240	1.69	57.8
LEFS	0.106	0.040	0.312	2.665	**.011**	0.026	0.186	1.51	75.1

R^2^ = 0.429, adjusted R^2^ = 0.364, F = 6.613, *P* < .001, Durbin–Watson **=** 1.574.

Abbreviations: BMI = Body Mass Index; VAS = Visual Analog Scale (pain); HADS-D = Hospital Anxiety and Depression Scale–Depression subscale; EQ-5D-5L VAS = EuroQol 5-Dimensions 5-Levels Visual Analog Scale; LEFS = Lower Extremity Functional Scale; B = partial regression coefficient, Beta = standardized regression coefficient, 95% CI = 95% confidence interval, VIF = Variance Inflation Factor.

a The bootstrap variable-selection procedure was performed using 5000 bootstrap samples. In each sample, the full set of candidate predictors was entered and the backward elimination procedure was repeated. Selection frequency represents the percentage of bootstrap samples in which the predictor was retained in the final model.

Values of *P* < .05 were accepted as significant and are marked in bold.

The bootstrap variable selection analysis showed that LEFS was the most frequently retained predictor, selected in 75.1% of bootstrap samples. EQ-5D-5L VAS and HADS-D were retained in 57.8% and 55.9% of samples, respectively. VAS pain was retained in 51.2% of samples, whereas BMI, HADS-A, and disease duration were selected less frequently (Table 4).

## Discussion

Sexuality is a key component of quality of life in individuals living with chronic diseases and plays a fundamental role in communication within intimate relationships.^29^ However, in chronic conditions such as lipedema, where physical symptoms are prominent, sexual difficulties are often left unspoken—neither openly expressed by patients nor adequately addressed during clinical assessments. Based on our knowledge, this study is the first in the literature to evaluate sexual function in women with lipedema using a valid and reliable quantitative tool, the FSFI, and to compare the results with an age- and BMI-matched healthy control group. Our findings showed that women with lipedema had significantly lower sexual function scores compared to controls and identified several factors associated with sexual function, including anxiety and depressive symptoms, lower extremity function, and pain intensity.

According to prevalence data, approximately 40% of women report experiencing sexual health problems.^30^ However, studies investigating the sexual experiences of individuals with lipedema remain limited. In a qualitative study by Dahlberg et al. women with lipedema were reported to avoid entering romantic relationships, which in turn reinforced avoidance behaviors regarding sexuality.^15^ Similarly, Falck et al. noted that pain, physical limitations, and difficulties in communication with partners contributed to reduced sexual desire and intimacy among women with lipedema.^31^ Melander et al. also demonstrated that internalized negative body perceptions had a markedly restrictive impact on the sexual lives of women with lipedema.^32^ In our study, 76% of women with lipedema had FSFI scores below the cutoff (≤ 26.55), indicating an increased risk of sexual dysfunction, compared to 36% in the healthy control group. Moreover, analysis of FSFI domains revealed that scores in desire, arousal, lubrication, orgasm, satisfaction, and pain were all significantly lower in the lipedema group compared to the control group. These findings suggest that various aspects of sexual functioning may be adversely affected in individuals with lipedema and highlight the potential value of incorporating sexual health assessment into clinical evaluation processes.

Lipedema is a chronic condition characterized not only by physical symptoms but also by significant psychological impacts.^33^ Psychological symptoms such as depression, anxiety, and social withdrawal have been reported to be more prevalent in individuals with lipedema than in the general population.^34^^,^^35^ In a recent study conducted in Czechia by Kunzová et al. more than half of women with lipedema were found to exhibit mild to moderate depressive symptoms, while 9.3% were classified as having severe depressive symptoms.^36^ Similarly, a retrospective cohort study conducted at a Swiss referral center involving 381 patients with lipedema reported that 23.4% screened positive for depression and 64.4% reported anxiety.^37^ Consistent with previous literature, our study found that women with lipedema had significantly higher levels of anxiety and depression than healthy women. Moreover, regression analysis showed that depression scores were independently associated with the total sexual function score. Correlation analyzes indicated that depression was significantly and negatively associated with sexual arousal, desire, and satisfaction domains. Anxiety levels also showed significant negative correlations with sexual satisfaction and pain. Depression, characterized by reduced motivation, diminished emotional engagement, loss of energy, and impaired capacity to experience pleasure, and anxiety, which is associated with heightened worry, performance concerns, and attentional shifts, may adversely affect multiple phases of the sexual response cycle.^38^ In particular, disruptions in anhedonia and reward-processing mechanisms may contribute to decreased sexual desire, while alterations in the perception of bodily sensations may interfere with arousal and sexual satisfaction.^39^ Indeed, studies in the general population have shown that depression may increase the prevalence of female sexual dysfunction by up to 70%-80%, with particularly pronounced impairments observed in the domains of desire, arousal, and orgasm.^40^ On the other hand, chronic pain and functional limitations commonly observed in patients with lipedema may contribute to the development or exacerbation of depressive and anxiety symptoms; meanwhile, psychological stress may increase symptom perception and weaken coping mechanisms. This bidirectional interaction may give rise to a self-sustaining cycle in which physical and psychological burdens mutually reinforce each other, ultimately leading to more pronounced impairments in sexual function over time. Considering these findings, integrating mental health support into the clinical care of women with lipedema may represent an important step toward enhancing emotional resilience and improving sexual function.

Another important finding of the study is the association of pain and lower extremity dysfunction with sexual functioning**.** In our study, significant negative correlations were found between pain levels and sexual function. Additionally, regression analysis showed that lower extremity functionality, as measured by the LEFS, was independently associated with sexual function. Indeed, previous studies have shown that in conditions such as fibromyalgia—a chronic pain syndrome characterized by heightened pain sensitivity—or musculoskeletal disorders like osteoarthritis, which can cause lower extremity pain and dysfunction, female sexual function is adversely affected.^41^^,^^42^ In the context of lipedema, reduced lower extremity function may directly limit movement, positioning, and physical comfort during sexual activity, thereby restricting participation and satisfaction. At the same time, chronic pain may increase attentional focus on bodily sensations and alter pain perception, leading to heightened anticipatory anxiety regarding potential discomfort during intimacy and triggering avoidance behaviors.^43^ Moreover, mobility limitations and persistent pain may reduce individuals’ participation in physical and social activities, contributing to physical deconditioning, reduced physical confidence, and increased psychological distress. Within this framework, sexual dysfunction may arise not only as a consequence of physical limitation but also as an outcome of cognitive-emotional processes and behavioral adaptations to chronic symptoms. Taken together, these findings suggest that structured exercise programs and multidisciplinary rehabilitation approaches aimed at reducing pain and improving functional capacity may have the potential to enhance not only physical health but also sexual functioning.

Health-related quality of life is a multidimensional concept that reflects the impact of an individual’s health status on their physical, psychological, social, and sexual dimensions.^44^ In a study conducted with patients diagnosed with lipedema, the majority of participants (87%) reported that the condition had a negative effect on their daily lives.^45^ Several studies have demonstrated that lipedema poses challenges not only in terms of physical burden but also by impairing emotional well-being and social participation, ultimately reducing overall quality of life.^46^^,^^47^ In our study, quality of life was also found to be significantly lower in women with lipedema compared to healthy controls. Additionally, a positive correlation was identified between EQ-5D-5L index scores and total FSFI scores.

Cultural factors may play an important role in shaping both the perception and reporting of sexual function.^48^ In sociocultural contexts such as Türkiye, where discussions around sexuality may be influenced by traditional norms, expectations of modesty, and potential stigma, women may be less likely to openly express sexual concerns or difficulties. This may lead to underreporting or altered interpretation of sexual experiences, thereby influencing the outcomes of self-reported measures such as the FSFI. In addition, gender roles, expectations within intimate relationships, and societal attitudes toward body image may influence the psychological and physical dimensions of sexual function in women with lipedema. Therefore, the findings of this study should be interpreted within a broader cultural context, and future studies are warranted to examine these findings across different cultural settings.

This study has several limitations. First, due to its cross-sectional design, causal inferences cannot be made regarding the associations between sexual function, anxiety and depressive symptoms, lower extremity function, and quality of life. Second, the relatively small sample size and recruitment from a single tertiary care center may limit the generalizability of the findings. Additionally, most of the data were collected through self-reported questionnaires, which may have introduced recall and response biases, particularly in sensitive areas such as sexuality. Furthermore, potentially influential variables such as relationship status, type of sexual activity (eg, partnered vs. solitary), partner-related factors, and hormonal status were not assessed in this study. Another important factor, body image, was also not evaluated and may be negatively affected in women with lipedema due to disease-related disproportionate fat distribution as well as associated esthetic and psychosocial burden, thereby indirectly influencing sexual function through its effects on self-perception and sexual confidence. In addition, although bootstrap variable selection analysis was performed to improve the robustness of the regression modeling procedure, several predictors demonstrated only moderate selection frequencies across bootstrap samples. This finding highlights the exploratory nature of the model and indicates that some uncertainty remains regarding the stability of the variable selection process. Therefore, the regression findings should be interpreted cautiously; future studies are recommended to employ more robust validation approaches, include additional psychosocial factors, and utilize larger, multicenter, and longitudinal designs.

## Conclusion

In conclusion, this study demonstrates that decreased sexual functioning is common among women with lipedema and that this may be associated with a variety of contributing factors. The findings indicate that the impact of lipedema extends beyond physical manifestations, with significant consequences for sexual health and overall well-being. Therefore, sexual *function* should not be overlooked in the clinical assessment of women with lipedema. A multidisciplinary and holistic treatment approach incorporating physical rehabilitation, pain management, psychological support targeting anxiety and depressive symptoms, and sexual health counseling may contribute to improved quality of life in this population.

## Data Availability

The datasets generated and/or analyzed during the current study are available from the corresponding author upon reasonable request.
